# Across species‐pool aggregation alters grassland productivity and diversity

**DOI:** 10.1002/ece3.2325

**Published:** 2016-07-22

**Authors:** Thomas P. McKenna, Kathryn A. Yurkonis

**Affiliations:** ^1^ Department of BiologyUniversity of North Dakota10 Cornell Street Stop 9019Grand ForksNorth Dakota58202

**Keywords:** Aggregation, biodiversity effect, complementarity effect, selection effect, tallgrass prairie

## Abstract

Plant performance is determined by the balance of intra‐ and interspecific neighbors within an individual's zone of influence. If individuals interact over smaller scales than the scales at which communities are measured, then altering neighborhood interactions may fundamentally affect community responses. These interactions can be altered by changing the number (species richness), abundances (species evenness), and positions (species pattern) of the resident plant species, and we aimed to test whether aggregating species at planting would alter effects of species richness and evenness on biomass production at a common scale of observation in grasslands. We varied plant species richness (2, 4, or 8 species and monocultures), evenness (0.64, 0.8, or 1.0), and pattern (planted randomly or aggregated in groups of four individuals) within 1 × 1 m plots established with transplants from a pool of 16 tallgrass prairie species and assessed plot‐scale biomass production and diversity over the first three growing seasons. As expected, more species‐rich plots produced more biomass by the end of the third growing season, an effect associated with a shift from selection to complementarity effects over time. Aggregating conspecifics at a 0.25‐m scale marginally reduced biomass production across all treatments and increased diversity in the most even plots, but did not alter biodiversity effects or richness–productivity relationships. Results support the hypothesis that fine‐scale species aggregation affects diversity by promoting species coexistence in this system. However, results indicate that inherent changes in species neighborhood relationships along grassland diversity gradients may only minimally affect community (meter) – scale responses among similarly designed biodiversity–ecosystem function studies. Given that species varied in their responses to local aggregation, it may be possible to use such species‐specific results to spatially design larger‐scale grassland communities to achieve desired diversity and productivity responses.

## Introduction

Although there has been a long‐standing interest in elucidating the mechanisms that contribute to grassland biodiversity–ecosystem function (BEF) relationships, it is still relatively unclear to what extent plant community responses are sensitive to variation in plant neighborhood composition. Plants exist in spatially limited neighborhoods that are defined by the distances over which individuals interact. If the scales of interaction among neighbors are sufficiently smaller than the scales over which communities are measured, then changing neighborhood interactions may affect community productivity and overall plant species diversity–productivity relationships (Lamošová et al. 2010; Zhang et al. 2014).

The effect of altering neighborhood composition on community productivity depends on the competitive relationships among species in the focal pool. When strong competitive differences occur among species in the pool, increasing the frequency of conspecific neighborships through aggregation may allow weaker competitors to persist as a result of delayed competitive exclusion (Stoll and Prati 2001; Monzeglio and Stoll 2005) and temporal priority effects (Porensky et al. 2012). This is well established theoretically (Chesson and Neuhauser 2002; Rácz et al. 2006) and at least experimentally within annual or newly establishing communities (Stoll and Prati 2001; Monzeglio and Stoll 2005; Porensky et al. 2012). In this case, if aggregation benefits less competitive and presumably less productive species, then aggregated communities would be less productive and more diverse than nonaggregated counterparts due to greater abundances of subordinate species.

Alternatively, when species benefit more from adjacency with heterospecifics than conspecifics, increasing the frequency of conspecific neighborships through aggregation may reduce the contribution of positive heterospecific effects to community‐scale responses (Naeem et al. 1999; Mokany et al. 2008). This advantage toward heterospecific neighbors can arise as a result of interspecific niche partitioning and facilitative interactions (complementarity effects) which have been shown to be increasingly important in driving community biomass production over time (Cardinale et al. 2007; Fargione et al. 2007). In this case, if aggregation reduces beneficial heterospecific interactions, then aggregated communities would be less productive and less diverse than nonaggregated counterparts because of a decrease in complementary interactions and a greater disparity in species abundances.

Within experimental settings, conspecific aggregation appears to affect species coexistence during grassland establishment (Porensky et al. 2012; Orwin et al. 2014; Yurkonis and McKenna 2014; Seahra et al. 2015), but it is unclear whether these effects are ubiquitous and persistent within increasingly diverse communities. To date, aggregation studies have mostly assessed aggregation effects at submeter scales over a single growing season (Monzeglio and Stoll 2005; Mokany et al. 2008; Orwin et al. 2014; Yurkonis and McKenna 2014). This limits our ability to assess how changing neighborhood relationships affect grasslands at common scales of observation (but see Yurkonis et al. 2012; Seahra et al. 2015) and prevents us from elucidating how species aggregation affects the development of grassland complementarity effects which typically arise after several growing seasons (Cardinale et al. 2007). Lamošová et al. (2010) and Zhang et al. (2014) are the only studies to date that have asked whether aggregation alters grassland diversity–productivity relationships. In both cases, conspecific aggregation reduced development of complementarity effects in the most species‐rich communities over a single season, but it is unclear whether these effects would persist within diverse, perennial grasslands.

Our goal was to ascertain how changes in neighborhood interspecific relationships affect diversity and productivity responses along perennial grassland richness and evenness gradients. We increased intraspecific interactions along richness and evenness gradients to determine how submeter neighborhood composition affects meter‐scale BEF relationships within a 3‐year manipulative field experiment with an extensive species pool. We test the hypotheses that conspecific aggregation reduces community biomass production either by promoting species coexistence and, thus, increasing diversity or by reducing niche partitioning and facilitative interactions (complementarity) and, thus, decreasing diversity. Findings help to elucidate the effect of changing neighborhood relationships on biomass production responses in BEF studies.

## Materials and Methods

### Experimental design

The Species Pattern and Community Ecology experiment consists of plots arranged in a randomized complete block design with five blocks established at the University of North Dakota's Mekinock Field Station (Lat 47.9620/Long −97.4517) in May 2012. Greenhouse grown transplants (16 weeks old) were planted into 1 × 1 m plots (2m spacing) divided into an 8 × 8 grid (64 individuals per plot). Plots varied in richness (2, 4, 8 species, and monocultures), evenness (0.64, 0.8, and 1), and species pattern (random or aggregated) (3 levels richness × 3 levels evenness × 2 levels pattern = 18 mixtures; (18 mixtures + 16 monocultures) × 5 blocks = 170 plots). Abundances at low, intermediate, and high evenness within each richness level were 8:56, 16:48, and 32:32 in two species plots, 4:4:28:28, 8:8:24:24, and 16 individuals per species in four species plots, 4:4:4:4:4:8:16:20, 4:4:4:4:12:12:12:12, and 8 individuals per species in eight species plots. Species were randomly allocated to the low, medium, and high abundances within evenness treatments. The pattern treatment was applied at the plot level, and each species was assigned independently (dispersed) to planting positions or to a group of four adjacent planting positions (aggregated) a plot. The site was in continuous agriculture for the previous 15 years, and a cultivator was used to remove weed seedlings prior to planting into bare soil. Soils at the site are moderately well drained LaDelle silt loam with 0–2% slopes. Transplants were watered as needed for 2 weeks to aid in plot establishment. Misplants and dead individuals were replaced during this 2‐week establishment period. Plot species composition was maintained with monthly weeding during the growing season, and aisles were mowed as needed.

The species composition of each plot was determined by randomly selecting species from a pool of 16 common tallgrass prairie species with four representatives from each functional group (warm and cool season grasses, forbs, and legumes). Species functional diversity was constrained as follows: two species plots contained a grass (warm or cool season) and a legume or a forb, four species plots contained one species from each functional group, and eight species plots contained two species from each functional group. The cool season grasses: *Pascopyrum smithii* (PS; western wheatgrass), *Elymus canadensis* (EC; Canada wildrye), *Elymus trachycaulus* (ET; slender wheatgrass), and *Nassella viridula* (NV; green needle grass), the warm‐season grasses: *Andropogon gerardii* (AG; big bluestem), *Panicum virgatum* (PV; switchgrass), *Schizachyrium scoparium* (SS; little bluestem), and *Sorghastrum nutans* (SN; Indian grass), the forbs: *Helianthus maximiliani* (HM; maximilian sunflower), *Monarda fistulosa* (MF; wild bergamot), *Ratibida columnifera* (RC; yellow coneflower), and *Solidago rigida* (SR; stiff goldenrod), the legumes: *Desmodium canadense* (DC: showy tick trefoil), *Astragalus Canadensis* (AC; Canada milkvetch), *Dalea purpurea* (DP; purple prairie clover), and *Glycyrrhiza lepidota* (GL; American licorice) were used in this experiment (seed obtained from Prairie Restorations Inc., Princeton, MN). Seed was stored at −20°C, and legume seeds were mixed with genus‐specific inoculant (Prairie Moon Nursery, Winona, MN) prior to seeding in the greenhouse.

### Data collection

During the first three growing seasons (May–August), soil surface light and soil moisture were recorded every 2 weeks. Above and below canopy photosynthetic active radiation (PAR) was recorded (AccuPAR LP‐80; Decagon Devices, Inc., Pullman, WA) between 10 am and 3 pm (daylight savings time) on cloudless days and used to calculate the proportion of available PAR reaching the soil surface. Percent volumetric soil moisture measurements (Scout TDR 100 with 20‐cm probes; Spectrum Technologies, Inc.; Aurora, IL) were 0.25 m made inside the north and south plot edges and averaged for each plot.

At the end of each growing season (September), aboveground biomass was cut to 5 cm above the soil surface, sorted to species, dried to a constant mass (60°C), and weighed. Plot Simpson's diversity (*D*) was calculated as $D=1/(\sum_{i=1}^{s}p_{i}^{2})$, where *S* is the number of species in the community and *p*_*i*_ is the proportional biomass of species *i*. Selection and complementarity effects were calculated using the additive‐partitioning model of Loreau and Hector (2001) based on species mixture and monoculture biomass production. This was based on species proportions by individuals (number of individuals of species *i*/64 individuals) at planting for year one and previous year proportion of plot biomass for each species in years two and three. Species proportions and species richness were adjusted (7% of plots) for persisting misplants not corrected in the establishment period. If a species not in the assigned species pool was planted (6% of plots), the individual was removed, and the total biomass for the plot was adjusted by adding the appropriate number of average individual weights for that species.

**Figure 1:**
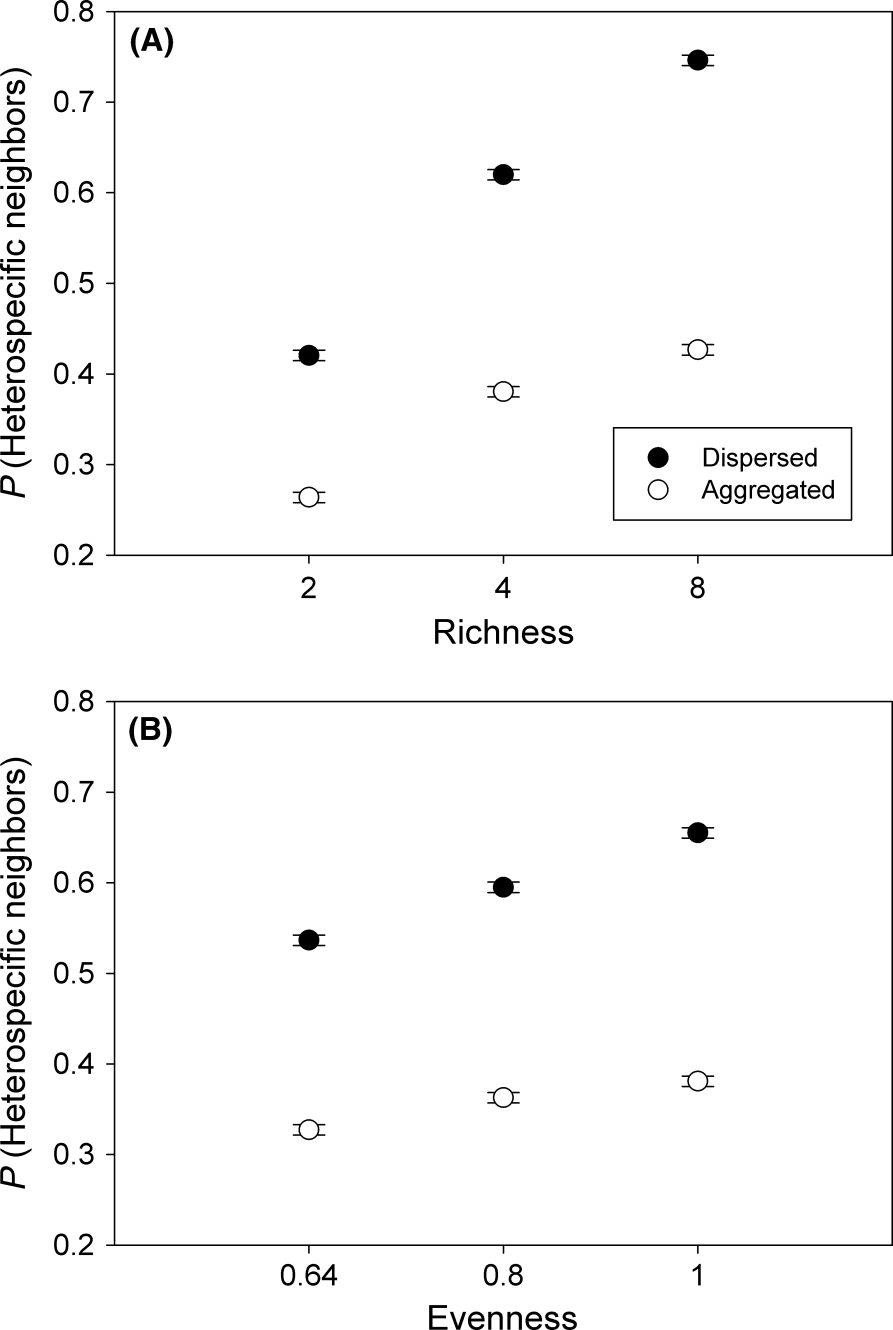
The proportion of all possible neighborships that occurred among heterospecific neighbors (mean ± SE) initially varied across plots planted at differing species richness (A) and evenness (B) levels. Plots were planted with individuals randomly assigned to 64 planting positions (dispersed) or in groups of four conspecific individuals (aggregated).

Plot interspecific interactions were quantified as the summed proportion of all possible neighborships that occurred among heterospecific neighbors. The program QRULE (Gardner and Urban 2007) was used to calculate species proportional neighborships for each initial planting map based on the closest neighbors for each individual (four neighbor rule with no diagonals). The proportion of heterospecific neighborships increased with increasing species richness (Fig. 1A) and species evenness (Fig. 1B), and in both instances, dispersed plots had greater heterospecific association than aggregated plots.

### Data analysis

Species richness, evenness, and pattern effects on biomass production, selection, and complementarity were compared across the three growing seasons with repeated‐measures ANOVA (proc mixed; SAS v9.3, Cary, NC) with fixed block effects. Soil surface light and volumetric soil moisture were similarly compared across sample dates within each growing season. Plot biomass, Simpson's Diversity, and soil moisture were natural log transformed, percent PAR was arcsine square root transformed, and selection and complementarity were square root transformed with the original sign maintained (Loreau and Hector 2001) to meet assumptions. Significant ANOVA tests were followed by least significant difference tests to identify differences among treatment groups.

Species‐specific performance in dispersed and aggregated plots was quantified by calculating per individual performance. This was calculated for each year by dividing the biomass of each species in a plot by the number of individuals originally planted of that species. Because of unequal and low sample size, an exact Wilcoxon two‐sample test (npar1way; SAS v9.3) was used to compare species differences between dispersed and aggregated plots within each year.

## Results

**Figure 2:**
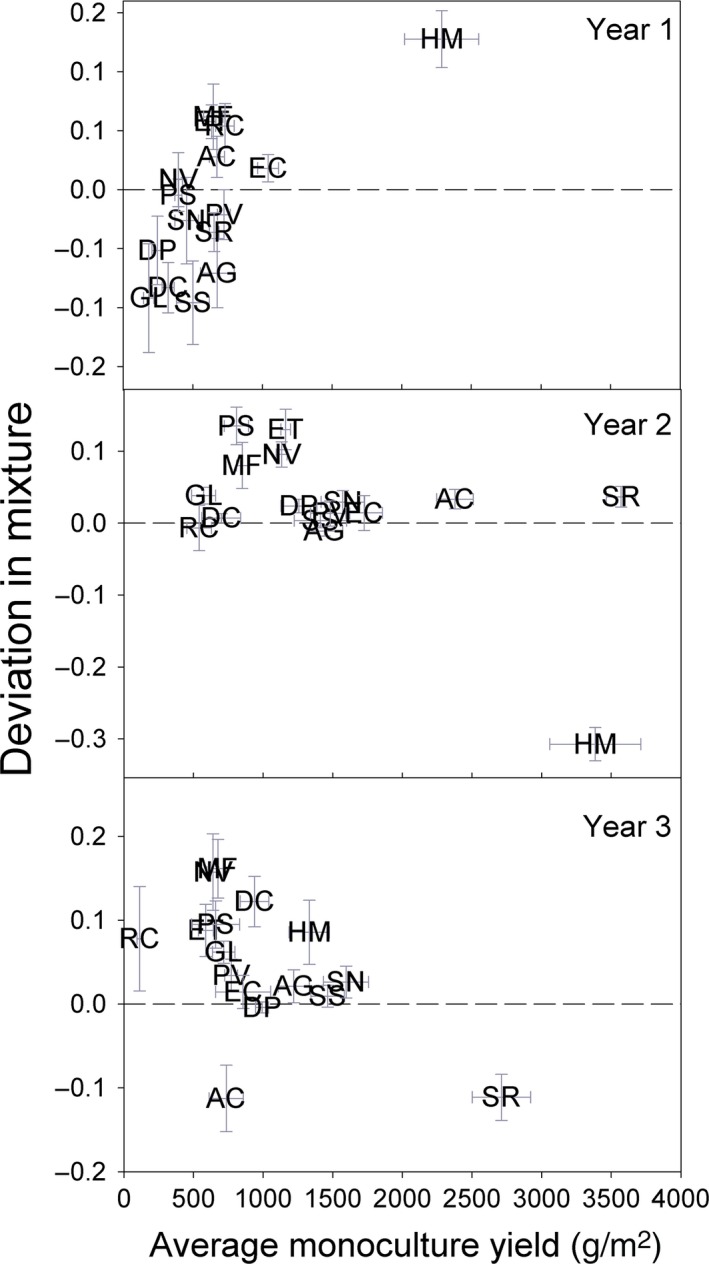
Difference in species relative yields (ΔRY = RY_O__bserved_− RY_E__xpected_) across all treatments (mean ± SE) and in relation to their monoculture yields (mean ± SE) in each growing season. Species labeled with the first letter of their genus and specific epithet: *Pascopyrum smithii* (PS), *Elymus canadensis* (EC), *Elymus trachycaulus* (ET), *Nassella viridula* (NV), *Andropogon gerardii* (AG), *Panicum virgatum* (PV), *Schizachyrium scoparium* (SS), *Sorghastrum nutans* (SN), *Helianthus maximiliani* (HM), *Monarda fistulosa* (MF), *Ratibida columnifera* (RC), *Solidago rigida* (SR), *Desmodium canadense* (DC), *Astragalus Canadensis* (AC), *Dalea purpurea* (DP), and *Glycyrrhiza lepidota* (GL).

Biomass production varied among species and over time (Fig. 2). The forbs *H. maximiliani* and *S. rigida* consistently produced the most aboveground biomass when grown solely in the presence of conspecifics (monoculture) while legumes produced the least (Fig. 2). Warm and cool season grasses were intermediate in their monoculture biomass production. In the presence of heterospecific effects (mixture), *M*. *fistulosa* and *R. columnifera* were the only species that consistently produced more biomass than would have been expected based on their monoculture performance (deviation in mixture; Fig. 2). Additionally, there was a temporal shift in the type of species that performed well in the presence of heterospecific effects. Species that produced less biomass in the presence of conspecifics (low monoculture yields) shifted from producing less biomass than expected to producing more biomass than expected in the presence of heterospecific effects (year one vs. year three; Fig. 2). In particular, all of the grasses overyielded in mixture relative to monoculture by the third year.

**Figure 3:**
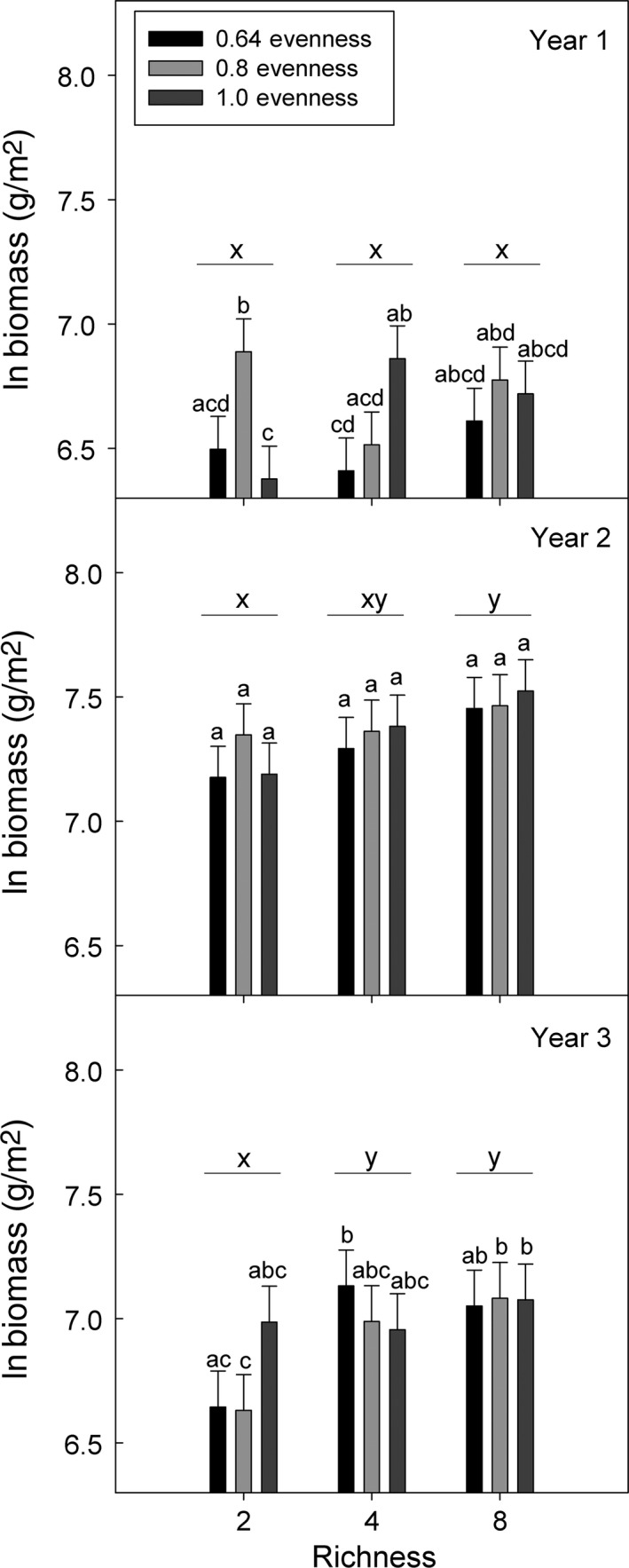
Effects of richness and evenness on aboveground biomass production (LS ln transformed mean ± SE) for each year of the experiment. Different letters above the lines (*x* and *y*) indicate differences among richness levels within year, and different letters above the bars (a–d) indicate differences within year among richness and evenness levels (LSD test).

**Figure 4:**
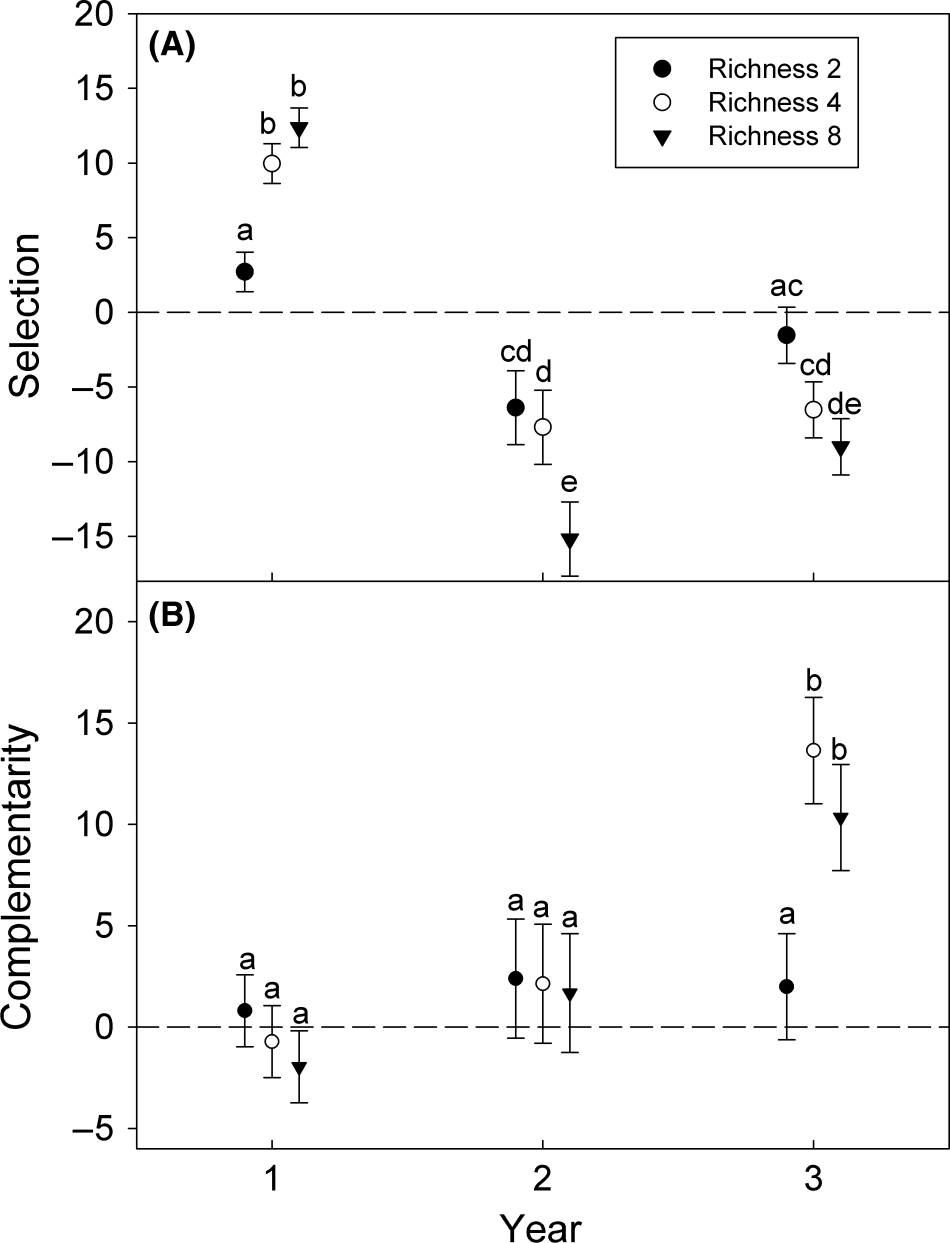
Selection effects (A) and complementarity effects (B) at each richness level for the first three growing seasons (LS square root transformed mean ± SE). Within each graph, different letters indicate a significant difference (LSD test).

As is common in BEF experiments, community‐scale biomass production was most strongly affected by the richness manipulation and was variable among years (Table 1, Fig. 3). Differences in biomass production among richness treatments were initially driven by selection effects, and selection effects increased with species richness (Fig. 4A). In years two and three, selection effects were negative and decreased with species richness. Selection also marginally differed between the most even (LS transformed mean ± SE = −1.12 ± 0.95) and intermediate (−4.20 ± 0.95) evenness plots. Complementarity effects developed within the four and eight species plots by the end of the third growing season (Fig. 4B), but these outcomes were not affected by evenness treatments.

**Table 1:** Results from repeated‐measures ANOVA of planted richness, evenness, and pattern effects on biomass production, biodiversity effects, and Simpson's Diversity within the first growing seasons

Effect	Biomass		Selection		Complementarity		Simpson's diversity	
	df	*F*	df	*F*	df	*F*	df	*F*
Block	4,68	0.55	4,68	0.97	4,68	3.84**	4,68	1.73
Richness (R)	2,67.9	3.15*	2,68	2.09	2,65.6	0.75	2,68.2	127.29**
Evenness (E)	2,67.9	0.65	2,68	2.91†	2,65.6	1.16	2,68.2	0.25
Pattern (P)	1,67.9	3.79†	1,68	0.00	1,65.6	1.32	1,68.2	2.69
R × E	4,67.9	0.28	4,68	0.56	4,65.6	1.52	4,68.2	2.04†
R × P	2,67.9	1.70	2,68	0.40	2,65.6	0.16	2,68.2	0.18
E × P	2,67.9	2.30	2,68	0.30	2,65.6	0.94	2,68.2	5.60**
R × E × P	4,67.9	0.85	4,68	0.31	4,65.6	1.13	4,68.2	2.34†
Year (Y)	2,71	363.47**	2,71	78.64**	2,71	17.43**	2,71	1.99
Y × R	4,84.4	1.67	4,84.4	9.10**	4,84.4	3.70**	4,84.4	1.37
Y × E	4,48.4	1.41	4,84.4	1.39	4,84.4	0.92	4,84.4	1.89
Y × P	2,71	0.07	2,71	1.33	2,71	0.60	2,71	2.92†
Y × R × E	8,98.5	3.04**	8,98.5	0.93	8,98.5	1.08	8,98.5	0.93
Y × R × P	4,84.4	0.76	4,84.4	0.89	4,48.4	1.25	4,84.4	0.81
Y × E × P	4,84.4	1.52	4,84.4	1.16	4,48.4	0.33	4,84.4	0.90
Y × R × E × P	8,98.5	1.16	8,98.5	0.66	8,98.5	1.53	8,98.5	1.60

Values are *F*‐statistics and degrees of freedom (df).

*P < 0.05, **P < 0.01, ^†^ *P* < 0.1.

**Figure 5:**
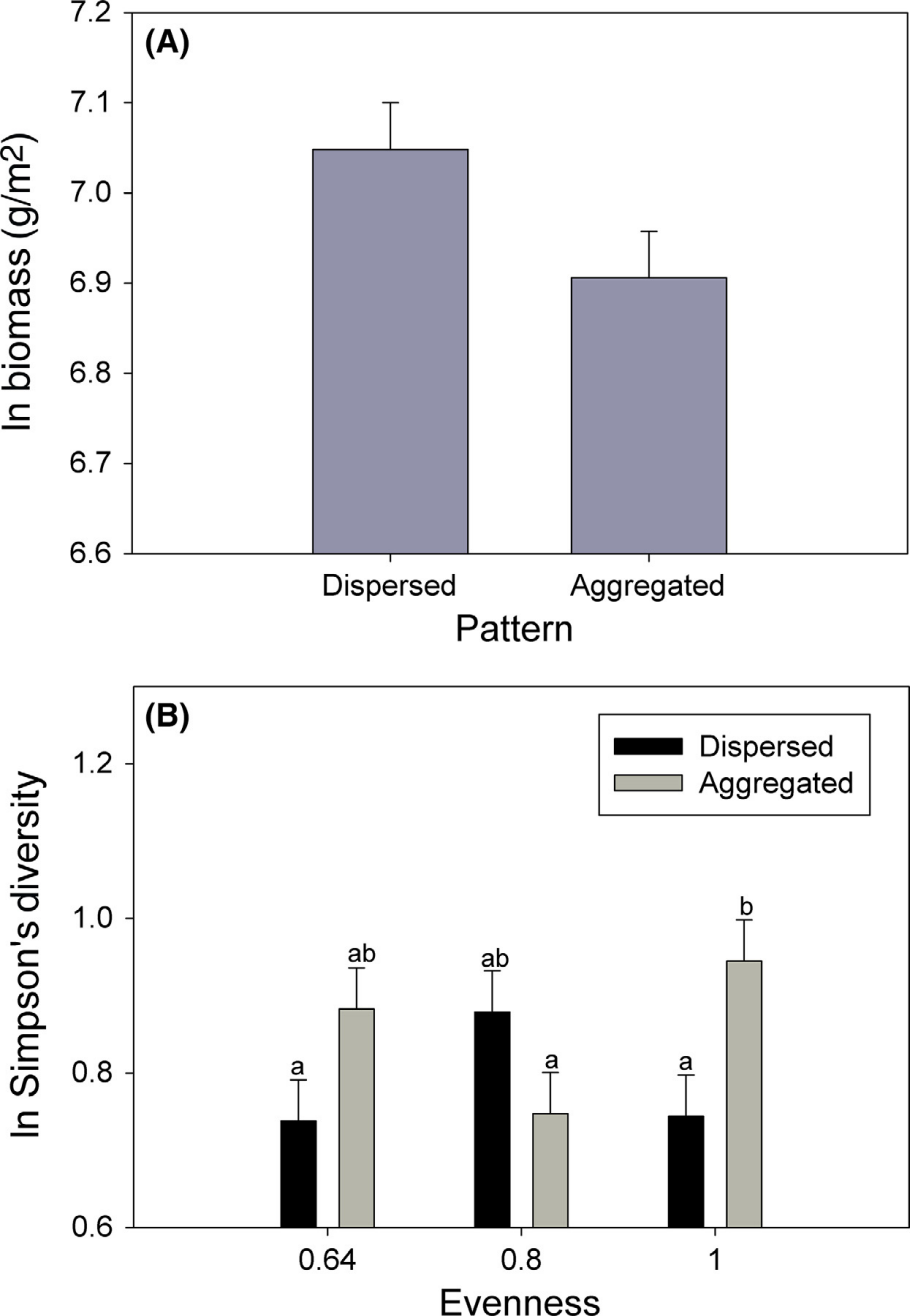
Effects of planted species pattern on aboveground biomass production (A; LS ln transformed mean ± SE) and effects of species pattern at each evenness level on Simpson's Diversity (B; LS ln transformed mean ± SE). Bars with different letters are significantly different (LSD test).

Species aggregation marginally affected biomass production and species diversity responses. Aggregated plots produced marginally less biomass than dispersed plots across richness and evenness treatments (Fig. 5A). Additionally, aggregated plots were more diverse in the most even treatment (Fig. 5B). Aggregation did not affect community‐scale selection or complementarity effects.

**Figure 6:**
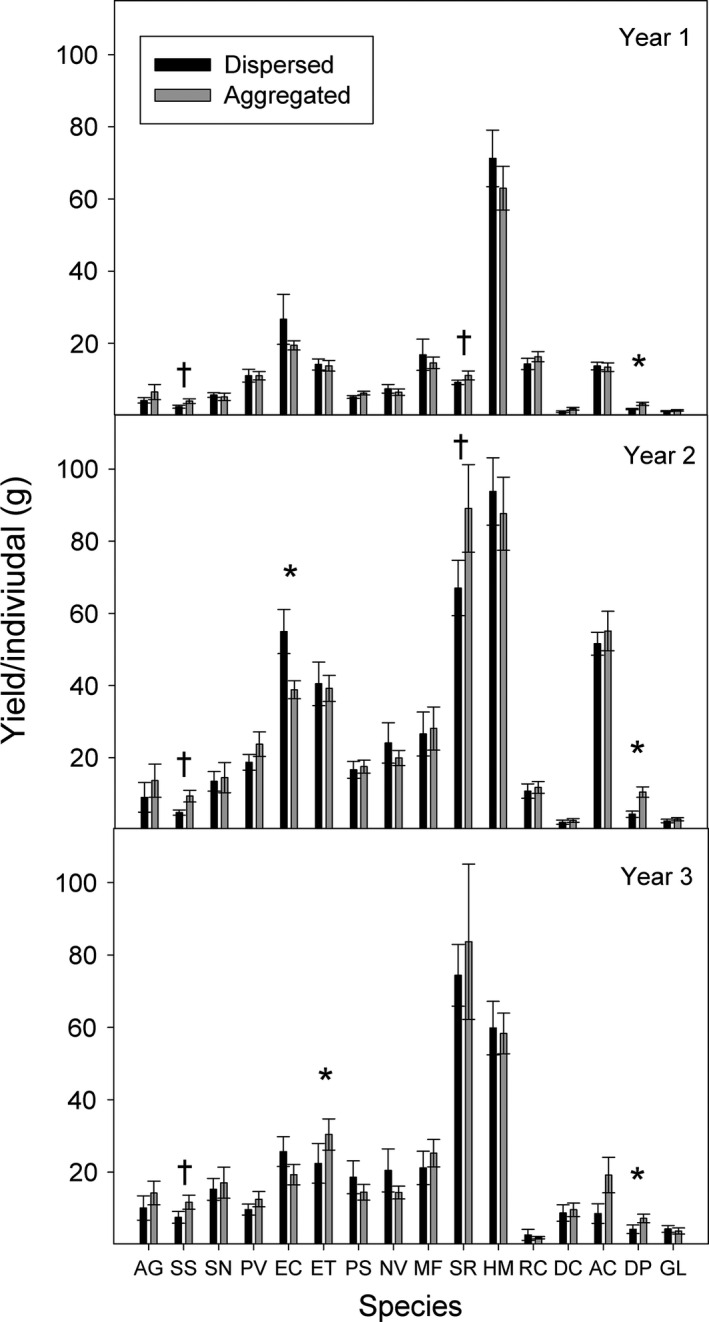
Yield per individual (mean ± SE) for each species in dispersed and aggregated plots for all 3 years of the experiment. An exact Wilcoxon two‐sample test was used to compare yields within species and year between pattern treatments (*P < 0.05, ^†^ *P* < 0.10).

Species varied in their responses to the aggregation treatment within mixed communities. In all 3 years, the per individual yield of *D. purpurea* (*P* < 0.05 for all 3 years) and *S. scoparium* (*P* < 0.10 for all 3 years) was greater in aggregated plots than dispersed plots (Fig. 6). *Solidago rigida* yielded marginally more per individual in aggregated plots than in dispersed plots in the first and second year (*P* < 0.10 in both years). *Elymus canadensis* yielded less per individual in aggregated plots than dispersed plots in the second year (*P* = 0.0248), and *E. trachycaulus* yielded more per individual in aggregated plots than in dispersed plots in the third year (*P* = 0.0399; Fig. 6).

Aggregation affected some measures of community‐scale resource use. Aggregated two species plots (0.80 ± 0.02%) intercepted less light than two species dispersed plots (0.74 ± 0.02%) in the first growing season (Richness × Pattern: *F*_2,65.6_ = 3.20, *P* = 0.0474). In early June of the third growing season, aggregated intermediate evenness plots intercepted marginally less light than dispersed counterparts, but this effect disappeared thereafter (Date × Evenness × Pattern: *F*_8,96.7_ = 2.29, *P* = 0.0271). Additionally, aggregated plots (LS transformed mean ± SE = 3.98 ± 0.021%) were marginally drier than dispersed plots (4.04 ± 0.021%; Pattern: *F*_1,65.4_ = 3.76, *P* = 0.0567) in the third growing season.

There were also treatment effects on resource use that were not affected by the pattern manipulation. In year one, early season effects of evenness in two species plots declined over the season and evenness effects developed over the season in four species plots (Date × Richness × Evenness: *F*_16,131_ = 2.17, *P* = 0.0089). In early July of the second season, there was an effect of evenness on soil moisture along the richness gradient (Date × Richness × Evenness: *F*_20,142_ = 2.08, *P* = 0.0072). In the third growing season, four and eight species plots intercepted more light than two species plots (Richness: *F*_2,63.2_ = 6.73, *P* = 0.0022).

## Discussion

We tested whether or not neighborhood conspecific aggregation affected community (meter)‐scale biomass production and diversity along richness and evenness gradients within a tallgrass prairie experimental system. As with previous studies, conspecific aggregation decreased (marginally) biomass production (Lamošová et al. 2010; Zhang et al. 2014) along richness and evenness gradients and increased diversity (Houseman 2013; Yurkonis and McKenna 2014) within the most even plots. While complementarity increased over time, these effects were not affected by aggregation at the 0.25‐m scale. Findings indicate that fine‐scale species aggregation decreased productivity by promoting species coexistence rather than by decreasing complementarity effects. Although submeter aggregation may contribute to initial diversity maintenance in this system, species aggregation and inherent changes in species spatial relationships along diversity gradients are not likely to substantially affect productivity and diversity responses in this setting.

Species varied in their responses to aggregation and more investigation is needed into the scales over which individual species interact and whether aggregation can be manipulated on a species‐by‐species basis to alter grassland productivity and diversity. Conspecific aggregation benefitted a select group of species and did not limit growth of the most productive species in this system. The only species hindered by aggregation was the most productive cool season grass in this study (*E. canadensis*), but this effect was limited to the second growing season. In contrast, four of the 16 species showed some evidence of improved yields with aggregation. This group included more and less productive species within each functional group. Most notably, *S. scoparium* and *D. purpurea* performed better in aggregated plots in all 3 years. For these species, aggregation may improve yields due to temporal dynamics that alter species access to light and spatial resources. For *S. rigida* and *E. trachycaulus*, the benefits of aggregation were temporally variable, which indicates that the outcomes of these species interactions with neighboring individuals are potentially context dependent.

After 3 years, there was no strong evidence that fine‐scale species pattern affected the development of grassland complementarity effects. Although the frequency of intra‐and interspecific interactions was substantially altered with our treatments, our 0.25‐m aggregation treatment was not sufficient enough to reduce community (meter)‐scale measures of niche partitioning and facilitation. This outcome likely arose as a majority of the species were interacting with neighboring individuals on scales larger than 0.25 m. Although we were unable to effectively isolate these species from heterospecific effects in mixtures, these species may need manipulations on larger scales (>0.25 m) to affect their yields and interactions with other species (Seahra et al. 2015).

Given that a majority of the species in the pool were not affected by submeter patterning and that there was little effect on species heterospecific interactions, it appears that species pattern changes along diversity gradients may only minimally affect diversity and productivity outcomes in similarly designed BEF studies. However, it may still be possible to affect diversity, productivity, and related community‐scale responses by manipulating neighborhood composition on a species basis and with attention to species neighborships (e.g., positioning of legumes relative to grasses). Additionally, such aggregation may alter other functions such as invasion (Yurkonis et al. 2012), root biomass production (Orwin et al. 2014), soil microbe community structure (Massaccesi et al. 2015), or insect interactions (Parachnowitsch et al. 2014) due to changes in the patterning and overall resource use. Future studies need to consider to what extent fine‐scale species pattern affects these processes and functions and to what extent our species‐specific results could be used to design spatially structured communities (e.g., in grassland reconstruction settings or within larger experimental plots) to achieve desired grassland diversity and productivity goals.

## Conflict of Interest

None declared.
